# Evolving Roles of Notch Signaling in Cortical Development

**DOI:** 10.3389/fnins.2022.844410

**Published:** 2022-03-29

**Authors:** Fang-Shin Nian, Pei-Shan Hou

**Affiliations:** ^1^Institute of Anatomy and Cell Biology, National Yang Ming Chiao Tung University, Taipei, Taiwan; ^2^Institute of Brain Science, College of Medicine, National Yang Ming Chiao Tung University, Taipei, Taiwan; ^3^Brain Research Center, National Yang Ming Chiao Tung University, Taipei, Taiwan

**Keywords:** Notch, cortical development, cortical evolution, neurogenesis, HES, DLL

## Abstract

Expansion of the neocortex is thought to pave the way toward acquisition of higher cognitive functions in mammals. The highly conserved Notch signaling pathway plays a crucial role in this process by regulating the size of the cortical progenitor pool, in part by controlling the balance between self-renewal and differentiation. In this review, we introduce the components of Notch signaling pathway as well as the different mode of molecular mechanisms, including *trans*- and *cis*-regulatory processes. We focused on the recent findings with regard to the expression pattern and levels in regulating neocortical formation in mammals and its interactions with other known signaling pathways, including Slit–Robo signaling and Shh signaling. Finally, we review the functions of Notch signaling pathway in different species as well as other developmental process, mainly somitogenesis, to discuss how modifications to the Notch signaling pathway can drive the evolution of the neocortex.

## General Introduction of Notch Signaling

Over a century ago, Morgan and Dexter identified hereditary mutant flies having wings with serrated edges (Morgan, 1911; Dexter, 1914) because of Notch deficiency (Morgan, 1917). Subsequently, studies have revealed that Notch and the corresponding signal pathways are highly conserved among species including *Drosophila melanogaster* (Go et al., 1998), *Caenorhabditis elegans* (Chen and Greenwald, 2004), *Lytechinus variegatus* (Sherwood and McClay, 1997), *Danio rerio* (Liao et al., 2016), and *Mus musculus* (Shimojo et al., 2008; Borrell et al., 2012; Cárdenas et al., 2018). Notch is involved in the regulation of cell fates in variable lineages (Artavanis-Tsakonas et al., 1999), cell survival, proliferation (Purow et al., 2005), and differentiation (Apelqvist et al., 1999) in a juxtacrine manner through the crosstalk between corresponding ligands and receptors.

**FIGURE 1:**
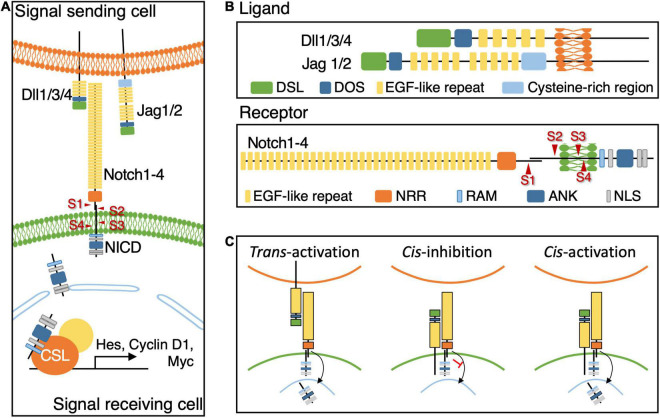
The components and Core Notch Signaling Pathway. **(A)** The illustration diagram of canonical Notch signal pathway showing the crosstalk between a ligand on the signal sending cells and a receptor on the signal receiving cell triggers the cleavage of Notch receptor to release NICD fragment. Nuclear-translocated NICD fragment interacts with CSL transcription factors to activate the downstream genes. **(B)** The schematic diagram showing the composition of Notch ligands, Dll1/3/4 and Jag1/2, and receptors, Notch 1–4 in mammals. Arrows indicate four protease cleavage cites on Notch receptors, S1–4. **(C)** The schematic diagram of different regulatory processes of Notch receptor-ligand interaction. The cell membrane marked in green represent the signal receiving cell.

Notch signaling, also known as the canonical Notch signaling pathway, is initiated through the interaction of a ligand on a signal-sending cell with a receptor on a signal-receiving cell (Figure 1A). The majority of Notch ligands and their receptors are single-pass type I transmembrane proteins with an intracellular C terminus and an extracellular N terminus (Figure 1B). Notch ligands contain the extracellular delta, serrate, and lag2 (DSL) domain that selects the corresponding receptors to mediate Notch activities (Kopan and Ilagan, 2009). Notch receptors contain extracellular epidermal growth factor (EGF)–like repeats that interact with the DSL domain of Notch ligands. The interaction triggers the cleavage of the intracellular Notch receptor to release the Notch intracellular domain (NICD) fragment. Subsequently, the NICD fragment is translocated into the nucleus to activate the downstream gene cascade by interacting with DNA-binding transcription factors such as CBF1, SU(H), and LAG1 (CSL) in vertebrates (Figure 1A). In addition, another non-canonical Notch signaling pathway has been uncovered in the recent two decades (Shawber et al., 1996; Nofziger et al., 1999; Bush et al., 2001). Unlike the canonical Notch signaling pathway, the non-canonical Notch signaling pathway activates Notch receptors independent of the DSL domain of Notch ligands or regulates downstream genes independent of CSL transcription factors (Andersen et al., 2012).

The structure of Notch ligands is critical in regulating the activity of canonical Notch signaling (Figure 1B). The extracellular N terminus contains several conserved domains including the DSL domain and EGF-like repeats. The DSL domain selects the corresponding subtype receptors, and EGF-like repeats determine the binding affinity to Notch receptors. Most of the Notch ligands possess a transmembrane domain at the C terminus, while some of them are not observed in *C. elegans*. On the basis of the absence or presence of cysteine-rich regions located between EGF-like repeats and the transmembrane domain, drosophila Notch ligands can be classified into two groups: Delta and Serrate. The vertebrate orthologs of Delta and Serrate are known as Delta-like and Jagged, respectively (Fleming, 1998). Mutagenesis analysis of subunits of Notch ligands revealed their roles in mediating Notch signaling, such as DSL domain (Henderson et al., 1994, 1997) or EGF-like repeats (Tax et al., 1994). In addition to the drosophila studies, missense mutant of Jagged1 induces Nodder (Hansson et al., 2010) and Slalom (Tsai et al., 2001) in mice and, in Human, mutations in DSL and EGF-like repeats domains of *JAG1* cause Alagille syndrome and mutations in only EGF-like repeats cause familial tetralogy of Fallot (Eldadah et al., 2001). Another highly conserved DOS domain (Delta and OSM-11-like proteins) sitting between the DSL and EGF-like repeats domains is known to cooperate with the DSL domain to facilitate Notch signaling (Komatsu et al., 2008), although it is missing in the majority of Notch ligand subtypes in *C. elegans*. Komatsu et al. (2008) found an OSM-11 protein carrying the DOS domain supports Notch ligands to activate Notch signaling during vulval development in *C. elegans*. Moreover, they demonstrated that the mammalian non-canonical Notch ligand Deltalike1 (Dlk1) can replace OSM-11 during the development of *C. elegans*, suggesting the presence of another mechanism that activates Notch signaling by using non-canonical ligands with the DOS domain, such as Dlk1/2, in invertebrates and vertebrates (Komatsu et al., 2008).

Notch receptors are type-1 transmembrane proteins (Figure 1B). In mammals, four paralogs of Notch receptors (Notch1–4) have been identified with similar structures but distinct corresponding ligands and functions. Mase et al. (2021) reported that the Notch1 receptor substantially maintains the radial glia (RG) pool during the early neurogenic stage of forebrain development, whereas Notch1 and 2 receptors contribute during the late stage. The extracellular domain of Notch receptors contains multiple EGF-like repeats that interact with Notch ligands and control the binding affinity. The negative regulatory region (NRR) adjacent to EGF-like repeats prevents the activation of the Notch receptor without binding to ligands. Intracellular Notch receptors contain a RBP-Jκ associated molecule (RAM), multiple ankyrin (ANK), and one-to-two nuclear location signal (NLS) domains. One of the NLS domain is located between the RAM and ANK domain and the other, if there is, is after the ANK domain (Lubman et al., 2007). RAM and ANK domains recruit transcription factors, and the NLS domain helps in their transportation into the nucleus. In addition, four proteolytic sites (S1–S4) are present between the intracellular and extracellular domains. S1 is cleaved by furin convertase to form the complete structure of the Notch receptor (Bray, 2006). S2 is located near the transmembrane domain on the extracellular side and is cleaved by ADAM metalloproteases. S3 and S4 are located in the transmembrane domain and would be cleaved by γ-secretase. Once Notch signaling is activated by the ligand–receptor interaction, S2 is first cleaved, followed by S3 and S4 (Figure 1). The cleavage releases NICD fragments containing RAM and ANK domains that translocate into the nucleus to control downstream target gene expression (Kopan and Ilagan, 2009).

Downstream target genes of Notch signaling include genes encoding the hairy and enhancer of split (Hes) protein family such as *E(spl)* genes in drosophila, *her1* and *hey1* in zebrafish, and *Hes1* and *Hes5* genes in mice (Jarriault et al., 1998). The cluster of Hes proteins belongs to the basic helix-loop-helix family. They function as transcriptional repressors to suppress differentiation genes, such as *Ngn2*, to retain the abilities of self-renewal and differentiation capacity (Tomita et al., 1999; Borrell et al., 2012). Moreover, Hes proteins may upregulate downstream genes such as the cell cycle regulator *Cyclin D1*, the upregulation of which would maintain cells in the cell cycle (Ronchini and Capobianco, 2001) and the protooncogene *cMyc* in cancer cells (Weng et al., 2006; Figure 1A).

Given the delicate and complex structure of Notch receptors and their ligands, Notch signaling is involved in various regulatory mechanisms. The extracellular calcium concentration affects Notch activity during left-right determination in vertebrates (Raya et al., 2004). This effect can be attributable to EGF-like domains in Notch receptors and their ligands that interact with calcium ions, which affect the ligand–receptor binding affinity (Rao et al., 1995; Cordle et al., 2008a,b). In support of this, the NRR in Notch receptors contains Ca^2+^-binding sites observed in the X-ray structure (Gordon et al., 2007). In contrast to the activation of Notch signaling by Notch ligands and receptors in adjacent cells (*trans*-activation), the interaction between Notch ligands and receptors within the same cell can inhibit Notch signaling (*cis*-inhibition) (Figure 1C). Although conflicts may occur in the binding sites of Notch receptors and their ligands during *trans*-activation and *cis*-inhibition, *trans*-activation and *cis*-inhibition can compete with each other (Cordle et al., 2008a). del Álamo et al. (2011) proposed that proteolytic sites responsible for generating NICD fragments are shed and that Notch signaling cannot be initiated when Notch ligands and their receptors are concurrently bound in the same cell through *cis*-inhibition (Figure 1). Because Notch ligands contain multiple proteolytic sites that can be either cleaved by ADAM metalloproteases or γ-secretases near the transmembrane domain (Zolkiewska, 2008), some Notch ligands appear to be soluble, even though they contain transmembrane domains, such as DeltaC in zebrafish and Dll3 in mammals (Geffers et al., 2007). Soluble Notch ligands may not be able to activate Notch signaling and instead act as an antagonist (Ladi et al., 2005; Chapman et al., 2011). This phenomenon might be induced by the binding of soluble Notch ligands to their corresponding Notch receptors in a *cis*-inhibitory conformation (D’souza et al., 2008); however, this regulatory mechanism is still under debate (Geffers et al., 2007). Beyond the classical concept of *trans*-activation and *cis*-inhibition (Sprinzak et al., 2010; LeBon et al., 2014); Nandagopal et al. (2019) demonstrated that *cis*-activation of Notch signaling can occur when the cell density was rigorously controlled *in vitro* (Figure 1C). They found Notch signaling can be activated in a cell which expressed both Notch ligands and receptors in the absence of surrounding cells. While this finding of *cis*-activation expends the possibility of regulatory mechanisms of Notch signaling, the related biological functions as well as the interaction with the conventional ways of *trans*-activation and *cis*-inhibition remained to be elucidated (Nandagopal et al., 2019). Thus, the phenotype induced by mutant Notch ligands lacking the C-terminus, including intracellular and transmembrane domains, might not be due to haploinsufficiency but dominant negative effects (Bulman et al., 2000; Warthen et al., 2006; Fischer-Zirnsak et al., 2019). However, Notch signaling mediates cell fate determination in variable cell types. Restricted combinations of ligand and receptors in canonical Notch signaling pathway may not be sufficient for all Notch-mediated developmental processes, suggesting an alternative pathway may be involved in. That might be the non-canonical Notch signaling pathway as conserved receptors are utilized, although detailed functions remain unclear (D’souza et al., 2010).

## Notch Signaling in Neocortex Formation in Mammals

At the early beginning of embryo development, the telencephalon originates from the most anterior part of the neural tube arising from a single layer of epithelial cells. On the basis of the anatomical position and composition of cell types, the telencephalon can be categorized into dorsal and ventral compartments. The neocortex, which is believed to be responsible for higher cognitive functions, is a major part of the dorsal telencephalon. The neocortex is formed by a six-layer laminated structure composed of glutamatergic excitatory neurons. Here, we focus on the involvement of the Notch signaling pathway in the formation of the laminated structure.

### Transition From Neuroepithelial Cells to Radial Glias

Distinct types of neurons in the neocortex are all derived from neural progenitor cells. Hence, the number of neural progenitor cells is critical to determine the size of the brain. The development of the neocortex begins with the generation and expansion of neural progenitor cells. In mammals, at least three types of neural progenitor cells are involved in the development of the neocortex: neuroepithelial cells (NECs), RGs, and intermediate progenitor cells (IPCs). NECs are the earliest type of neural progenitor cells that are highly polarized in a pseudostratified pattern (His, 1889; Ramon y Cajal and Azoulay, 1955). Because NECs are believed to generate all other types of cells in the neocortex, the size of the NEC pool is crucial to determine the numbers of progenitor cells and even the final number of cortical neurons (Malatesta et al., 2000; Noctor et al., 2001, 2002). To amplify their pool, NECs keep dividing symmetrically and exponentially before the onset of neurogenesis. NECs gradually transform into RGs for the onset of neurogenesis. Although RGs still maintain some NEC characteristics, such as bipolar morphology and apical–basal polarity (Rakic, 1972), they begin to lose tight junctions (Aaku-Saraste et al., 1996) and express specific RG proteins (Levitt and Rakic, 1980), such as glutamate/aspartate transporter (Shibata et al., 1997) and brain lipid-binding proteins (Feng et al., 1994). Although RGs could symmetrically divide to expand its pool as NECs, they can undergo asymmetrical division to produce neurons. In addition to their self-renewal and differentiation functions, the radial fiber of RGs guides neuronal migration. In this process, the overexpression of cleaved NICD fragments promote progenitor cells to express RG-specific markers (Gaiano et al., 2000). No differences in the number of NECs in the neural tube were observed between *Hes1/5* double-knockout mice and control mice at the NEC stage E8.5, whereas the number of RGs decreased due to prematuration at later stages (E9.5–10.5) when NECs begin to transform to RGs (Hatakeyama et al., 2004). These studies suggest that the transition of NECs to RGs is dependent on Notch signaling, whereas the formation and expansion of NECs is independent of Notch signaling.

### Generating Intermediate Progenitors or Neurons From Radial Glia

Neurogenesis from RGs to neurons can occur in a direct or an indirect manner. Direct neurogenesis is one RG divides to generate an RG and a neuron in the ventricular zone (VZ), and indirect neurogenesis is one RG may generate two RGs or two other types of progenitor cells, such as IPCs. Subsequently, IPCs symmetrically divide to generate two neurons. Indirect neurogenesis is beneficial for the increase in the final neuron pool and is more common in the mammalian neocortex compared with direct neurogenesis, which is the predominant neurogenesis manner in the developing cortex of other vertebrates, such as birds and reptiles (Englund et al., 2005; Guillemot, 2005; Cárdenas et al., 2018).

Prematuration is observed in animal models with a Notch signaling deficiency. The aforementioned studies have indicated that defects in the activation of Notch signaling inhibited the transition from NECs to RGs. Because Mind bomb 1 (Mib1), a RING-type E3 ubiquitin ligase, promotes the endocytosis of canonical Notch ligands, knocking out the *Mib1* gene can impair Notch signaling. Conventional *Mib1* knockout mice exhibited deficient Notch signaling that led to prematuration at E9.0–E9.5, resulting in the death of embryos before E12.5 (Koo et al., 2005). Furthermore, in animal models with Notch signaling deficiency, RGs transformed into IPCs early before differentiating into neurons. In *Nestin*-driven *Mib1* knockout mice, the numbers of IPCs and mitotic cells outside the VZ region were increased at E13.5 (Yoon et al., 2008), resulting in an increase in the number of neurons from E14.0. Those findings suggest that Notch signaling activity is high in RGs but low in IPCs and neurons. To determine the activity of Notch signaling in RGs and IPCs separately, overexpression of *NICD* together with CBF1-EGFP, a reporter of Notch signaling, was utilized. The results revealed that NICD activated the *CBF1*-binding site in RGs but not in IPCs. Because NICD cannot activate Notch signaling in IPCs, Hes proteins can be overexpressed as an alternative method to activate Notch signaling. However, the numbers of IPCs decreased when Hes proteins were overexpressed (Mizutani et al., 2007; Ohtsuka and Kageyama, 2021b); this finding is in contrast to that of knockout experiments that indicated the attenuation of Notch activity. However, the reason underlying the inactivation of Notch signaling in IPCs remains to be elucidated. Because IPCs mediate indirect neurogenesis to effectively increase cell numbers and emergence of IPCs is crucial in the evolution of the mammalian neocortex (Cárdenas et al., 2018), the evolution of the mammalian neocortex should be examined by investigating the functional roles and molecular mechanisms of IPCs.

In gyrencephalic species, such as ferret and primates, a large population of proliferative cells can be noted in the basal region of the VZ. They are a subtype of RGs, called basal RGs (bRGs). These bRGs, unlike IPCs, have radial fibers but lose the apical attachment to the ventricular surface, unlike their apical cohorts, apical RGs (aRGs). bRGs can undergo self-renewal to expand the progenitor pool in the SVZ region. In the developing primate neocortex, the majority of bRGs are positioned in the outer SVZ (OSVZ), which is separated from the inner SVZ (ISVZ) by an inner fiber layer. During neocortical expansion, the thickness of the OSVZ gradually increases with the expansion of the VZ (Rakic, 1974; Smart et al., 2002; Lukaszewicz et al., 2005; Lui et al., 2011). Except for the similarity in morphological characteristics between bRG and aRGs, bRGs express some aRG genes, such as SOX2, PAX6, nestin, and GFAP, and undergo a Notch signaling–dependent pathway to self-renew or generate IPCs in the OSVZ (Fietz et al., 2010; Hansen et al., 2010). The induction of radial glial fiber divergence in the superficial neocortex by a large number of bRGs produced through the basal process combined with neuronal migration along the newly formed fibers can cause lateral dispersion and promote cortical folding in gyrencephalic species (Reillo et al., 2011; Gertz and Kriegstein, 2015; Llinares-Benadero and Borrell, 2019). Moreover, because of the abundant generation of bRGs and their daughter cells, the OSVZ was determined to be the predominant neurogenic zone at the mid-gestational stage that caused marked cortical neuronal expansion and an increase in brain size in humans, thus leading to the evolution of the cerebral cortex (Hansen et al., 2010; Lui et al., 2011; Llinares-Benadero and Borrell, 2019).

### Oscillation Pattern of Notch Signaling in Neural Progenitor Cells

In the last decade, a group led by Professor Ryoichiro Kageyama in Japan published a series of discoveries describing several components in the Notch signaling pathway are expressed in a dynamic pattern called oscillation, which has been reported earlier and is essential in somitogenesis (Palmeirim et al., 1997). They found that the oscillation of *Hes1* can maintain the pool of neural progenitor cells. Concurrently, the expression of Notch ligand *Dll1* and the proneural gene *Ngn2* were fluctuated in a manner which is coordinated but opposite to the oscillated expression pattern of *Hes1*. The fine balance of the oscillating gene expression pattern is orchestrated by several elaborate transcriptional regulatory mechanisms. The oscillating pattern of *Hes1* expression can be regulated through a negative feedback loop. After the activation of *Hes1* by the Notch ligand–receptor interaction, Hes1 protein cis-represses its own transcription by directly targeting its promoter. Another key is the short half-life of *Hes1* mRNA and Hes1 protein. The half-life of *Hes1* mRNA and Hes1 protein is as short as 20 min. As both *Hes1* mRNA and Hes1 protein are degraded soon after their production, the *Hes1* promoter can be released from autoinhibition. Also Hes protein represses proneural genes such as *Mash1* (Chen et al., 1997) and the expression of *Dll1* is directly regulated by Ngn2 and Mash1 through the regulation of enhancer regulatory elements (Castro et al., 2006), the oscillated pattern of *Dll1* and proneuronal genes *Ngn2* and *Mash1* are similar to and follow that of *Hes1* (Shimojo et al., 2008; Imayoshi et al., 2013). Nonetheless, the oscillating *Ngn2* expression remains to be validated because previous findings have indicated that most cells, if not all, of *Neurogenin2 CreER* and *R26R-CAG-loxPstop-EGFP* mice had left the progenitor pool at 12 h after tamoxifen administration (Miyoshi and Fishell, 2012). Thus, the oscillating *Dll1* expression pattern should be the most critical event in orchestrating *Hes1* expression and Mash1 may be the upstream activator of *Dll1* instead of Ngn2 (Imayoshi et al., 2013; Sueda et al., 2019). Interestingly, while the *Hes* genes oscillated in multiple tissues across species, the frequency varies. For instance, during somitogenesis when the oscillated *Her/Hes* expression regulated the formation of new somite, the frequency differs in different species: 30 min in zebrafish, 90 min in chick, 2 h in mouse (Cinquin, 2007), and 4–6 h in humans (Turnpenny et al., 2007; Kageyama et al., 2012; Hubaud and Pourquié, 2014; Matsuda et al., 2020). The period of *Hes1* oscillation in mouse neural progenitor cells and fibroblasts is 2 h. However, the period is 3–5 h in mES cells (Kobayashi et al., 2009; Kobayashi and Kageyama, 2011), suggesting that the period may vary among cell types as well as the regulatory machinery. If the oscillation of *Hes1* can maintain the pool of neural progenitor cells, the neuronal production step in neurogenesis indicates the escape of the oscillation cycle. Hence, neuronal differentiation can be induced by the sustained *Ngn2* expression in the replacement of oscillatory *Ngn2* expression (Shimojo et al., 2008). However, in this scheme, how *Ngn2* and *Dll1* expression escape the negative feedback loop controlled by Hes1 and changes from the oscillatory pattern to a sustained high expression pattern remain unclear.

The oscillatory Hes1 expression can be used to maintain neural progenitors in the cell cycle, whereas sustained Hes1 expression promotes cells to stay in a quiescent state (Sang et al., 2008; Sueda et al., 2019) that may contribute to boundary formation such as the boundary between the dorsal and ventral telencephalon (Baek et al., 2006). The sustained overexpression of *Hes1* in mouse neural progenitor cells at E13.5 reduced the expression of Notch ligands (*Dll1* and *Jag1*), proneural genes (*Mash1* and *Ngn2*), and cell cycle regulators (*cyclin D1* and *cyclin E1*) (Shimojo et al., 2008; Sueda et al., 2019). This result suggested that the sustained overexpression of Hes1 repressed both proliferation and differentiation. Thus, cells in the boundaries of the brain were not able to proliferate or differentiate. In *Hes1*-overexpressing transgenic mice, Pax6+/Hes1+ neural progenitor cells were maintained for a long time in the VZ even after birth. Nonetheless, compared with control mice, *Hes1*-overexpressing mice exhibited the suppressed proliferation of abnormal neural progenitor cells and a markedly elongated cell cycle length; this finding is in agreement with the previous study indicating that the sustained overexpression of Hes1 reduced the expression of cell cycle–related proteins such as *cyclin D*1 (Shimojo et al., 2008). Further investigation using transgenic mice to engineered wild-type *Hes1* gene into the shortened or elongated form found both amplitude and frequency of oscillated *Hes1* expression were impaired which resulted in neural prematuration and reduced brain size (Ochi et al., 2020) similar to the phenotype induced by engineered *Dll1* gene (Shimojo et al., 2016). Notably, the shortened or elongated form of *Dll1* gene would cause the deficiency in both neural development and somite formation. In addition to manipulating the pattern of oscillation, the basal level of *Hes1* expression is also critical to its biological functions. Contrary to the mutant *Hes1* mice expressing reduced as well as sustained levels of Hes1, overexpression of Hes1 prevented neural progenitor cells from self-renewal and differentiation, thus leading to a smaller brain size, a thinner cerebral cortex, the enlarged ventricles in *Hes1*-overexpressing mice and an apparent increase in the number of neural progenitor cells even in the late corticogenesis (Ohtsuka and Kageyama, 2021b). However, another interpretation has been raised by Borrell et al. (2012) proposing that Hes1 expression is crucial to maintaining the progenitor cell pool in the VZ by overexpressing *Hes1* cDNA or downregulating *Hes1* expression by using the RNA interference (RNAi) technique. Another study showed that activation of Notch signaling maintains the neural progenitor cell pool by overexpressing the NICD fragment (Mizutani et al., 2007). Thus, whether maintaining the neural progenitor pool is controlled by simply activation of Notch signaling or in the combination of the oscillated Hes1 expression remains to be clarified.

## Combinational Effects of Notch Signaling and Other Signaling Pathways in the Developing Brain

### Slit–Robo Signaling

Robo signaling is a widely known pathway involved in neural development. Robos and Slits (ligands of Robo receptors) are responsible for regulating axon guidance which contributes to cortical circuits (Brose et al., 1999; Dickson and Gilestro, 2006). Moreover, Robo–slit signaling regulates neurogenesis in the central nervous system (CNS) of drosophila (Mehta and Bhat, 2001) and mice (Andrews et al., 2008). In the neocortex of *Robo1/2* knockout mouse, neural progenitor cells in the VZ underwent a premature asymmetric division and increased the generation of IPCs, thus reducing the brain size. This deficiency was found to be mediated by Robo-mediated transcriptional activation of the Notch effector *Hes1*, which suggested the interplay between Robo and Notch signaling is crucial to regulate neurogenesis precisely (Borrell et al., 2012).

CNS evolution across species has been investigated for decades; however, it still remains largely unclear. The differential regulation of direct and indirect neurogenesis in different species is one of the most prominent hypotheses. Recently, a study examined the switch between Dll1–Notch and Robo–Slit signaling in corticogenesis to determine the predominant mode of indirect or direct neurogenesis along with its effects on the neuron number, brain size, and neural circuit complexity across amniotes. To elucidate the involvement of Notch–Dll1 and Robo–Slit signaling, the expression level in the neural progenitors of each representative species among diverse amniotes (snake, chick, mouse, and human) was analyzed. Data indicated a high Robo expression level and a low Dll1 expression level in brain structures including the dorsal telencephalon of snake, the medial dorsal telencephalon of chick, and the olfactory bulb, hippocampus, and spinal cord of mammals, but a high Dll1 expression level and a low Robo expression level in the advanced brain structures including the lateral dorsal telencephalon of chick and the neocortex of mammals (Cárdenas et al., 2018; Cárdenas and Borrell, 2020). In brief, Robo expression declined in the evolutionary process, whereas Dll1 expression increased during the evolution of amniotes. Furthermore, the combined gain-of-function of Dll1 and loss-of-function of Robo in the evolutionarily old region of the telencephalon in mouse, chick, and snake indicated the promotion of indirect neurogenesis. By contrast, the combined gain-of-function of Robo and loss-of-function of Dll1 in the evolutionarily young region of the telencephalon including the mouse neocortex and human cerebral organoids indicated the promotion of direct neurogenesis. This observation is correlated to the switch between direct and indirect neurogenesis. Progenitors in the snake dorsal cortex exhibit mostly direct neurogenesis with no indirect neurogenesis, as indicated by the absence of IPCs. By contrast, progenitors in the mammalian neocortex exhibit indirect neurogenesis most frequently. These findings were further confirmed in human organoids, indicating that the Robo–Dll reciprocal expression–based balance of direct/indirect neurogenesis is the key factor for evolution among amniotes (Cárdenas et al., 2018; Cárdenas and Borrell, 2020).

### Sonic Hedgehog Signaling

Sonic hedgehog (Shh) is a secreted protein encoded by *Shh* gene. Initially, *hedgehog* gene was identified from *Drosophila melanogaster*. Mutations in *hedgehog* gene lead to abnormal segmental patterning and polarity in flies (Nüsslein-Volhard and Wieschaus, 1980; Mohler, 1988). Shh signaling is essential for embryonic development in two stages. In the early stage, Shh is secreted from the notochord, located ventrally to the neural tube, and controls the neural axis by creating a concentration gradient (Echelard et al., 1993; Roelink et al., 1995). In the later stage, Shh regulates cell proliferation and differentiation during brain development by controlling cell cycle kinetics in various tissues and species such as the mouse neocortex (Bertrand and Dahmane, 2006; Komada et al., 2008; Komada, 2012) and chick spinal cord (Saade et al., 2013). Shh is essential to the development of IPCs (Shikata et al., 2011). Mutations in human *SHH* gene cause holoprosencephaly (HPE), which is an autosomal dominantly inherited disorder. Patients with HPE have intellectual disability, microcephaly, and epilepsy (Tekendo-Ngongang et al., 1993; Belloni et al., 1996; Roessler et al., 1996). Shh protein initiates signaling by binding to the transmembrane receptor Pathed (*Ptch)*, which inhibits Smoothened (*Smo*) in the absence of Shh (Murone et al., 1999). When Smo is de-repressed, it causes Gli1-3 to move to the nucleus, thus inducing downstream gene expression (Wickström et al., 2013).

Ohtsuka and Kageyama (2021b) reported that the sustained overexpression of Hes1 in mice retained abnormal neural progenitors with both Pax6 and Hes1 expression in the VZ even after birth but still accompanied by smaller brains, thinner cerebral cortices, and enlarged ventricles due to defects in proliferation and neurogenesis. Later, Ohtsuka and Kageyama (2021a) observed that *Hes1*-overexpressing mice could be rescued from their defects by crossing them with transgenic mice expressing constitutively active Smo, an effector of Shh signaling. This result suggested that dysfunction in Notch signaling can be complemented by promoting Shh signaling (Ohtsuka and Kageyama, 2021a). However, as both the pathways are crucial during embryonic development, detailed molecular mechanisms through which they work together in parallel or complement remain to be elucidated.

## Evolutionary and Comparative Perspectives

### Pallial Organization and Evolution in Vertebrates

The cortex of most of the reptiles such as alligators, geckos, and turtles shows a mixed pattern of the layered structure in the dorsal pallium dorsal to the ventricles and nuclear structures in the dorsal ventricular ridge ventral to the ventricles (Goffinet et al., 1986; Suzuki and Hirata, 2014; Briscoe and Ragsdale, 2018a,b; Nomura et al., 2020). In addition to different cytoarchitecture, neurons in the layered structure of reptiles migrate and integrate into the cortex roughly through an outside–in migration pattern (Suzuki and Hirata, 2014; Luzzati, 2015; Tosches et al., 2018; Nomura et al., 2020), opposite to the inside–out migration pattern in the developing mammalian neocortex. The pallium of birds is composed of four major subdivisions: hyperpallium, mesopallium, nidopallium, and arcopallium (Jarvis, 2009). The pallium in birds and some reptiles have a nuclear-type structure, in which neuronal cell bodies aggregate instead of layered laminated structures such as the neocortex in mammals. On the basis of trajectory tracing and *in situ* hybridization analyses, recent studies have identified that neurons with similar functions and molecular expression across the species have a nuclear or laminar structure, regardless of different cytoarchitectures (Zeier and Karten, 1971; Karten and Shimizu, 1989; Dugas-Ford et al., 2012; Suzuki et al., 2012; Chen et al., 2013). For example, neurons in the L2 field of the cortex in zebra finches receive signals from the thalamus and express genes such as *Rorβ*, similar to layer IV sensory neurons in the mammalian neocortex; neurons in the mesopallium and nidopallium and neurons in the arcopallium exhibit conserved projections and molecular expression similar to layer II–III and layer V–VI neurons in the mammalian neocortex, respectively (Chen et al., 2013). Considering conserved functions and the phylogenic tree, the laminated structure should be evolved from the nuclear type. A nuclear-to-layered hypothesis proposed by Karten indicated that the laminated pallium of the mammalian neocortex might be transformed from the nuclear type pallium in birds or reptiles (Karten, 1991).

Comparative analysis of neuroanatomical structures, gene expression profiles, and neural circuits is a common approach used to study pallium formation in teleosts (Wullimann and Mueller, 2004; Wullimann, 2009). The structure of the pallium shows distinct morphological features in different teleosts. However, mechanisms underlying the development of the teleost cortex and the gene expression profiles of neuronal connections remain largely unknown. The teleosts are close to land vertebrates such as amphibians and reptiles in evolution, and can be divided into ray-finned fishes and lobe-finned fishes. A ray-finned fish, called zebrafish, is the most common animal model used to study embryonic development, diseases, and neurological behaviors. Accumulating results of *in situ* hybridization, immunostaining, and neural circuit tracing indicate that molecular profiles and presumptive functions in the pallium and subpallium of the teleostean cortex are similar to those of other vertebrates. For example, *Emx* genes are enriched in the pallium and *Dlx* genes are enriched in the subpallium across species (Wullimann and Mueller, 2004; Wullimann, 2009). These genetic studies suggested that pallium formation from the neural tube in ray-finned fishes follows a special method called “eversion,” in which the neural tube bends outward to form two cerebral hemispheres, separated by an unpaired ventricle and covered with a thin roof plate. In contrast to ray-finned fishes, the pallium of other vertebrates, such as lobe-finned fishes, amphibians, reptiles, birds, and mammals, is generated during an evagination process, in which the roof of the neural tube is sunken down to separate two lateral ventricles (Huesa et al., 2009; Yamamoto et al., 2017). The two prominent differences between these processes in terms of morphological changes are the inverted mediolateral axis in the pallium and the position of ventricles. Because the lumen surface of the neural tube is critical to generating neural progenitor cells, changing the position of ventricles may cause alterations in the direction of the neuronal migration and orientation of neural fibers (Huesa et al., 2009; Wullimann, 2009; Yamamoto et al., 2017).

In fishes and mammals, considerable changes have been observed in the neuron number, pallium cytoarchitecture, and neural circuit complexity. Because components in Notch signaling are highly conserved, the activity of Notch signaling may be widely involved in multiple developmental events in the formation of the pallium such as the maintenance of neural and cortical progenitor cell pools, transition from aRGs to IPCs, and corticogenesis. Thus, we speculate that the evolutionary divergence in pallium formation may result from the dominant isoform switch, the distinct regulation mechanism, or the emergence of novel genes, which will be discussed in the following sections.

### Dominant Isoform Switch: Comparison of Dll1/3 in Mammals and DeltaC/D in Zebrafish

**FIGURE 2:**
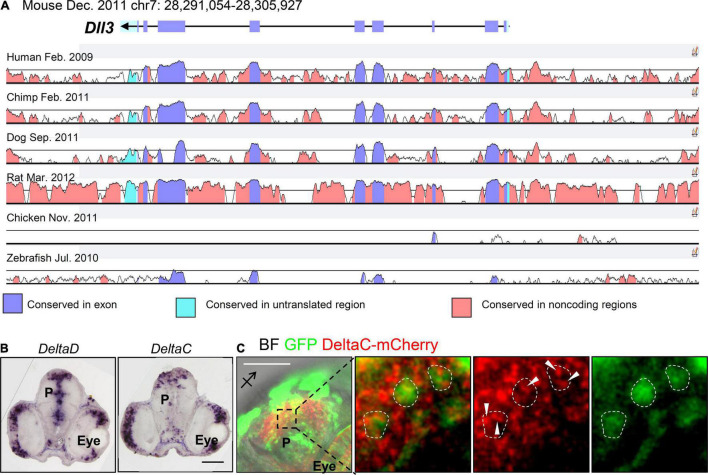
Conservation of Dll3 among species. **(A)** VISTA Browser display of a 14.9 kb fragment of the *Dll3* region on Chr. 7 of the mouse genome (chr7: 28,291,054–28,305,927). VISTA plots are shown in the seven-way (Mouse-Human-Chimp-Dog-Rat-Chicken-Zebrafish) alignment. Based on the annotation, conserved regions above 50%/100 bp in VISTA are above the cutoff, and are colored under the curve with the indicated color. **(B)** Spatial expression of *DeltaD* and *DeltaC* in the 2 dpf zebrafish brain slice. **(C)** The expression pattern of DeltaC–mCherry in the zebrafish brain 2 days after introducing the plasmids carrying *DeltaC–mCherry* or *GFP* at 4-cell stage. BF: bright field. White dotted lines circle GFP-positive cells and arrows indicate puncta pattern of DeltaC-mCherry. Scale bar: 100 μm.

In zebrafish, Notch ligands in the Delta family include DeltaA, DeltaB, DeltaD, and DeltaC, whereas Delta-like ligands include Dll1, Dll3, and Dll4 in mammals. DeltaA–D are expressed in the developing zebrafish pallium (Smithers et al., 2000; Mueller and Wullimann, 2003; Takano et al., 2011), whereas Dll1 and Dll3 (Nelson et al., 2013) but not Dll4 are expressed in the developing mammalian pallium (Herman et al., 2018). Comparing the DNA sequences of these delta genes with mouse *Dll1* and *Dll3* revealed that the sequences of *DeltaD*, *DeltaA*, and *DeltaB* are similar to that of mouse *Dll1*, whereas the sequence of *DeltaC* is similar to that of mouse *Dll3* (Figure 2A). As fewer studies have examined the roles of Delta proteins in the development of the zebrafish pallium, we would like to briefly introduce the functions of *Delta* genes during somitogenesis and elaborate their possible implications in pallial development.

In zebrafish, somitogenesis is controlled by segmentation clock, which is coordinated by several components in Notch signaling including a Notch ligand (*DeltaC*) and the downstream target hairy/E(spl) genes (*her1* and *her7*) with an oscillatory expression pattern. Both *DeltaD* and *DeltaC* are essential for somitogenesis, and *DeltaC*, as one of the oscillators, is critical for proper somite segmentation (Mara et al., 2007). Unexpectedly, a study using *DeltaD* mutant embryos suggested that DeltaD is required for the oscillation of *her1*, the downstream target gene of Notch signaling, whereas the expression of *DeltaD* is maintained at a constant level (Holley et al., 2000). These findings suggest that *DeltaC* and *DeltaD* expressed in different patterns have distinct functions during somitogenesis, and the deficiency of any protein causes defects in somite formation (Holley et al., 2000; Mara et al., 2007). Further examination by Wright et al. (2011) revealed the puncta expression pattern of DeltaC and DeltaD in the retina and hindbrain, and, notably, DeltaC and DeltaD were colocalized in the retina but not in the hindbrain. In cellular level, DeltaD may be either expressed in the cytoplasm or on the plasma membrane depending on the expression level of DeltaC in the presomitic mesoderm (PSM) during the formation of new somite (Wright et al., 2011). Mechanically, DeltaC is expressed as a soluble form to physically attract DeltaD away from the cell membrane to switch off DeltaD-mediated Notch signaling in the DeltaC-enriched region (Wright et al., 2011). In the developing pallium, *DeltaD* and *DeltaC* are both expressed, as demonstrated by our *in situ* hybridization data (*DeltaD* and *DeltaC*, Figure 2B) and previous studies (Smithers et al., 2000; Takano et al., 2011) and, in protein level, DeltaC was expressed in a puncta pattern in the cytoplasm, as shown by the DeltaC–mCherry strategy (Figure 2C). Considering the interplay between DeltaC and DeltaD in somitogenesis, DeltaC and DeltaD may play similar roles in regulating Notch signaling to control pallial formation.

During mammalian neocortical development, *Dll1* has been found to be expressed in neural progenitors with an oscillatory pattern (Shimojo et al., 2008). *In situ* hybridization in the E9.5 whole mount embryo indicated that *Dll1* was expressed in the forebrain, whereas *Dll3* was expressed only in the ventral region of the forebrain. During somite formation, *Dll1* and *Dll3* were differentially expressed in the posterior or anterior region of newly formed somite (Dunwoodie et al., 1997) and were both necessary for somitogenesis (Kusumi et al., 1998; Dunwoodie et al., 2002). Mutations in human *DLL1* induce neurodevelopmental disorders with non-specific brain abnormalities (Fischer-Zirnsak et al., 2019), whereas mutations in *DLL3* cause spondylocostal dysostosis with axial skeletal defects (Bulman et al., 2000). These pathological findings suggest the critical role of Dll1 in dorsal telencephalic development while Dll3 mainly functions in somitogenesis. The use of *Dll3* cDNA to replace *Dll1* gene resulted in embryonic lethality in transgenic mice, suggesting that at least some Dll1 functions cannot be replaced by Dll3 (Geffers et al., 2007). Besides, Dll1 was found on the plasma membrane, whereas Dll3 was observed in the cytosol with a puncta pattern in mouse PSM and cultured cell lines (Geffers et al., 2007; Chapman et al., 2011); this is similar to the distribution of DeltaC and DetlaD in zebrafish somitogenesis. Another reason to explain the interchangeable role of Dll1 by Dll3 is the absence of lysine within the intracellular domain of Dll3. As lysine in the intracellular domain would be ubiquitinated by the ubiquitin ligase, Mib1, to triggers the endocytosis to recycle the ligand on the signal sending cell and pulling Notch receptor on the signal sending cell to activate downstream signaling through exposing the S2 protease site (Ladi et al., 2005; Le Borgne et al., 2005; Sprinzak and Blacklow, 2021). Thus, Dll3 cannot be exhibited on the cell membrane to compensate the loss of Dll1. Besides the intracellular domain, Geffers et al. (2007) provided evidences showing N-terminal DSL domain and the first two EGF-like repeats of Dll1 were critical to activate Notch signaling and cannot be replaced by that of Dll3 using different forms of chimeric Dll1 and Dll3 fusion proteins. Komatsu et al. (2008) also reported that in addition to the DSL domain, the conserved DOS motif within the first two EGF-like repeats is vital for activating Notch signaling and suggested that the DOS motif may cooperate with the DSL domain in binding to the Notch receptor. Separate studies performing mutation and structural analysis have indicated the importance of the DOS motif in cell lines (Shimizu et al., 1999; Geffers et al., 2007; Cordle et al., 2008a) and *C. elegans* (Komatsu et al., 2008). However, mouse Dll3 and Dll4 and zebrafish DeltaC do not contain this DOS motif, which may explain why Dll3 is unable to activate Notch signaling in certain cell types (Ladi et al., 2005; Geffers et al., 2007). Although Komatsu et al. (2008) suggested that Notch ligands without the DOS motif, such as DeltaC and Dll3, may trigger non-canonical Notch signaling with non-canonical ligands with the DOS motif, the role of non-canonical Notch signal pathway in either neurogenesis or somitogenesis should be further confirmed.

Both zebrafish DeltaC and mouse Dll3 share some similar features such as the intracellular distribution and lack of a DOS motif. Mutation of either *DeltaD* or *DeltaC* in zebrafish would lead to defects in somite development (Holley et al., 2000; Mara et al., 2007), suggesting that *DeltaD* and *DeltaC* are both necessary for somitogenesis. Studies on human disorders have indicated that *DLL1* is more crucial for the neocortical development (Fischer-Zirnsak et al., 2019), whereas *DLL3* is more critical for somitogenesis (Bulman et al., 2000). Thus, during somite formation, the dominant isoform changes from *DeltaD* and *DeltaC* in zebrafish to *DLL3* in humans. This may reflect an evolutionary change in dominant forms in distinct tissue development. As *DeltaA–D* are all expressed in the developing zebrafish pallium (Smithers et al., 2000; Mueller and Wullimann, 2003; Takano et al., 2011), the expression pattern of *Delta-like* genes in mice and clinical features of human diseases suggest that the compensation may occur in the developing zebrafish pallium but not in the developing human dorsal telencephalon. These findings imply that the dominant form regulating telencephalic development may switch during the course of evolution.

### Distinct Regulatory Machinery Leads to Diverse Cortex Formation Among Species

**FIGURE 3:**
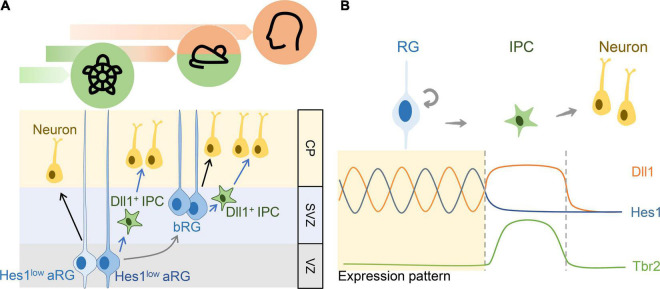
The role of Notch signaling in the balance between self-renewal and differentiation in the neural progenitors. **(A)** The schematic diagram showing the direct (black arrows) or indirect modes (blue arrows) of neurogenesis and the expression of Notch signaling components in the developing turtle, mouse, and human cortex. **(B)** The schematic diagram showing the dynamic expression pattern of key factors, Hes1 and Dll1, in Notch signaling and IPC gene Tbr2. The upregulation of Tbr2 follows the sustained Dll1 expression and the downregulation of Hes1 expression.

Cortical development involves multiple neural and cortical progenitors to produce cortical neurons at the right place and correct time. After the onset of corticogenesis, aRGs derived from RGs produce neurons either through the direct or indirect pathway (Figure 3A, black arrows: direct pathway; green arrows: indirect pathway). In the indirect pathway, aRGs generate to IPCs before producing neurons. Promotion of the indirect neurogenic pathway may be an evolutionary event (Cárdenas et al., 2018; Cárdenas and Borrell, 2020). A comparative approach using multiple species such as snakes and the mammalian pallium demonstrated the dominance of direct neurogenesis, whereas indirect neurogenesis gradually replaces direct neurogenesis in higher animals such as mammals (Figure 3A). Borrell’s team identified this evolutionary trend and suggested its relation to the gradient expression of Robo/Dll1 in the pallium across different species (Borrell and Reillo, 2012; Borrell et al., 2012; Cárdenas and Borrell, 2020). During indirect neurogenesis, RGs generate IPCs before becoming neurons, and one IPC symmetrically divides again to generate two neurons (Miyata et al., 2004; Noctor et al., 2004). IPCs act as a source of Notch ligands (Mizutani et al., 2007) to maintain the RG cell pool in a feedback loop of Notch signaling (Kawaguchi et al., 2008; Yoon et al., 2008; Lui et al., 2011; Nelson et al., 2013). Kawaguchi demonstrated that Dll1-positive cells in the VZ/SVZ of the E13.5 mouse neocortex were separated from those with active Notch signaling, and conditional *Dll1* knockout mice driven by *Nestin*-Cre showed neuronal prematuration, suggesting that Dll1 can maintain neural progenitors in an undifferentiated state (Kawaguchi et al., 2008). Yoon et al. (2008) used *Mib1* knockout mice in their study. Mib1 is a RING-type E3 ubiquitin ligase that promotes the endocytosis of canonical Notch ligands. They demonstrated that Mib1-positive cells may provide the Dll1 ligand to activate Notch signaling in adjacent cells *in vitro*. In addition, most Mib1-positive cells including IPCs and neurons can serve as Dll1 sources to activate Notch signaling in surrounding RGs (Yoon et al., 2008) which was supported by the asymmetric distribution of Dll1 and Mib1 during the asymmetric division of a neural progenitor to produce a progenitor and a neuron (Tozer et al., 2017).

Nelson et al. (2013) categorized the major expression of *Dll1* into two clusters in the SVZ and VZ at the E14.5 mouse neocortex similar to that reported in the Allen Brain Atlas^1^ (Figure 3A). The *in situ* hybridization results from the Allen Brain Atlas showed that *Dll1* was expressed in the VZ and SVZ but not in the cortical plate in the cortices at various developmental stages. In addition, the number of *Dll1*-expressing cells gradually decreased over the course of development. Through multiphoton microscopy, Nelson discovered that Dll1-positive IPCs could contact Hes1/5-positive RGs through dynamic and transient elongate processes to maintain the RG cell pool. In addition, they suggested that other Notch ligands may participate in Notch signaling such as Dll3 in IPCs and Jag1 in RGs (Nelson et al., 2013). On the basis of the findings of these studies, we summarized that the oscillatory expression of Notch ligands such as Dll1 or Jag1 (Nelson et al., 2013) and receptors (Notch1/2) (Mase et al., 2021) can maintain RGs in the cell cycle (Figure 3). After differentiation into IPCs, Notch ligands are expressed at a constant level because Notch signaling cannot be activated to produce downstream *Hes1*, which represses *Dll1* expression in a negative feedback loop (Figure 3B; Mizutani et al., 2007). With a constant expression of Dll1, IPCs can act as a Dll1 source to activate Notch signaling in RGs through ligand–receptor interaction. Oscillated *Hes1* and *Dll1* may occur only in RGs but not in IPCs. Although the detailed mechanism remains unclear, Notch signaling is believed to be inactivated in IPCs due to the lack of Notch receptors or the presence of molecules that inhibit the activation of Notch signaling. Finally, the expression of Dll1 is the lowest in neurons (Figure 3B). In the brains, such as reptiles and birds, which lack IPCs because they primarily rely on direct neurogenesis during pallium development, Notch ligands (Dll1 or Jag1) are produced solely by RGs. However, Notch ligands in RGs are oscillatory in response to the negative regulator Hes protein, which may explain the smaller RG pool in reptiles than in mammals due to the lack of an alternative source of Notch ligands (Figure 3A). During the development of the neocortex in humans, a significant increase in the number and types of neural/cortical progenitors contribute to a larger size of the dorsal telencephalon than in other vertebrates. The enriched bRG is the most prominent feature for massive cortical expansion. bRG is derived from aRGs similar to the derivation of IPCs from aRGs. Similar to direct and indirect neurogenic pathways, bRG may produce neurons directly or generate basal IPC before producing neurons. Notch signaling is activated in bRGs based on the expression of Hes1. In addition, according to Nelson’s study with Dll1d2YFP reporter, many neural/cortical progenitors, including RGs, bRGs and IPs, express Dll1, and basal IPCs can maintain bRG proliferation through physical contact with bRGs in the SVZ (Figure 3B; Nelson et al., 2013; Govindan and Jabaudon, 2017).

Nomura found that neural progenitors in the pallium of geckos required a long time to differentiate into neurons compared with other species of amniotes (Nomura et al., 2013). They applied pulse labeling to monitor the period from neural progenitors to neurons in the pallium of mouse (*M. musculus*), gecko (*Poekilocerus pictus*), turtle (*Pelodiscus sinensis*), and chick (*Gallus gallus*). Compared with the neural progenitors of other species, neural progenitors in geckos required a longer period to differentiate, twice as those required by mice and chicks. Although cortical progenitors stay in the progenitor stage for a longer period in geckos than in mice, the number of mitotic cells was lower in geckos, suggesting that the size of the neural progenitor pool may not be associated with the duration in the progenitor stage. Furthermore, they used a CBF1-driven reporter to monitor Notch activity in neural progenitors. Neural progenitors of geckos exhibited higher Notch activity than those of other species. Notably, the distribution of neural progenitors with active Notch signaling differed among species: mosaic in mice, turtles, and chicks but homogenous in geckos (Nomura et al., 2013). Two possibilities can explain the mosaic distribution of neural/cortical progenitors. First, this distribution may result from the oscillated expression pattern of Notch signaling components (Shimojo et al., 2008; Ohtsuka and Kageyama, 2021b). Second, this pattern may be due to scattered IPCs in the neural progenitor pool to deliver the Notch ligands (Mizutani et al., 2007). In either possibility, the cross-species study suggested that differences in the duration of neural progenitor differentiation and the number of mitotic cells within amniotes may be linked to the spatial distribution of neural/cortical progenitor cells, which may be uniform or mosaic. Thus, the emergence of the mosaic distribution of neural/cortical progenitor may be an evolutionary key to the expansion of the telencephalon.

### Novel Human-Specific Genes

Notch signaling is essential for self-renewal in RGs to maintain the progenitor cell pool during cortical development. Expansion of the neural progenitor pool and a prolonged neural progenitor self-renewal period are believed to be critical events in cerebral cortex evolution (Hansen et al., 2010; Lui et al., 2011; Borrell and Reillo, 2012; Geschwind and Rakic, 2013). Recently, a human-specific *NOTCH2* partial duplicated paralog, *NOTCH2NL* gene, was found to be expressed in both human aRG and bRGs and to improve the expansion of cortical progenitors by activating NOTCH signaling through interrupting the cis-inhibition of DLL1 (Suzuki et al., 2018). Overexpression of *NOTCH2NL* in embryonic mice or human organoids prolonged the self-renewal stage and delayed neuronal differentiation, resulting in clonal expansion in neural progenitors (Fiddes et al., 2018; Suzuki et al., 2018). By contrast, *NOTCH2NL* knockout accelerated neuronal differentiation and reduced neurogenesis (Fiddes et al., 2018). Investigations on underlying mechanisms revealed that NOTCH2NLs would interact with NOTCH receptors and inhibit cell autonomous DLL1 (NOTCH ligand) function to enhance NOTCH activity during corticogenesis (Fiddes et al., 2018; Suzuki et al., 2018). In addition, the deletion or duplication of *NOTCH2NL* genes in humans induced microcephaly and megacephaly, respectively, suggesting the crucial role of NOTCH2NL in human neocortical development (Fiddes et al., 2018). Hence, enhancing NOTCH signaling at a proper level may contribute to cortical evolution.

## Conclusion and Future Perspectives

Notch signaling is highly conserved among species and regulates a wide range of developmental processes. It had been demonstrated that the activity of the canonical Notch signaling pathway determines the size of the neural progenitor pool and the initiation of neural differentiation during the telencephalon development in amniotes (Nomura et al., 2013; Cárdenas et al., 2018). However, it remains unclear how Notch signaling contributes to the formation of the telencephalon in anamniotes and how the conserved Notch signaling contributes to the establishment of distinct telencephalic cytoarchitecture in different species. To facilitate the multiple roles of Notch signaling, it may utilize different combinations of ligands (Nelson et al., 2013) and receptors (Mase et al., 2021), interact with other signaling amniotes (Cárdenas et al., 2018; Cárdenas and Borrell, 2020; Ohtsuka and Kageyama, 2021a) or novel genes (Fiddes et al., 2018; Suzuki et al., 2018). Further, the involvement of non-canonical Notch signaling would improve the complex regulations by Notch signaling in orchestrated multiple developmental processes. These evidences demonstrate the delicate regulation of Notch signaling is capable of activating distinct downstream machinery in either the developmental processes or evolution. Thus, to delineate the whole pictures of Notch signaling is believed to decode the mystery underlying the brain evolution to acquire higher cognitive functions in mammals.

## Author Contributions

F-SN and P-SH collected the relevant research for the review and wrote the manuscript. Both authors contributed to the article and approved the submitted version.

## Conflict of Interest

The authors declare that the research was conducted in the absence of any commercial or financial relationships that could be construed as a potential conflict of interest.

## Publisher’s Note

All claims expressed in this article are solely those of the authors and do not necessarily represent those of their affiliated organizations, or those of the publisher, the editors and the reviewers. Any product that may be evaluated in this article, or claim that may be made by its manufacturer, is not guaranteed or endorsed by the publisher.

## Footnotes

1
https://portal.brain-map.org/

## References

[B1] Aaku-Saraste E. Hellwig A. Huttner W. B. (1996). Loss of occludin and functional tight junctions, but not ZO-1, during neural tube closure–remodeling of the neuroepithelium prior to neurogenesis. *Dev. Biol.* 180 664–679. 10.1006/dbio.1996.0336 8954735

[B2] Andersen P. Uosaki H. Shenje L. T. Kwon C. (2012). Non-canonical notch signaling: emerging role and mechanism. *Trends Cell Biol.* 22 257–265. 10.1016/j.tcb.2012.02.003 22397947 PMC3348455

[B3] Andrews W. Barber M. Hernadez-Miranda L. R. Xian J. Rakic S. Sundaresan V. (2008). The role of Slit-Robo signaling in the generation, migration and morphological differentiation of cortical interneurons. *Dev. Biol.* 313 648–658. 10.1016/j.ydbio.2007.10.052 18054781

[B4] Apelqvist Å Li H. Sommer L. Beatus P. Anderson D. J. Honjo T. (1999). notch signalling controls pancreatic cell differentiation. *Nature* 400 877–881. 10.1038/23716 10476967

[B5] Artavanis-Tsakonas S. Rand M. D. Lake R. J. (1999). Notch signaling: cell fate control and signal integration in development. *Science* 284 770–776. 10.1126/science.284.5415.770 10221902

[B6] Baek J. H. Hatakeyama J. Sakamoto S. Ohtsuka T. Kageyama R. (2006). Persistent and high levels of Hes1 expression regulate boundary formation in the developing central nervous system. *Development* 133 2467–2476. 10.1242/dev.02403 16728479

[B7] Belloni E. Muenke M. Roessler E. Traverse G. Siegel-Bartelt J. Frumkin A. (1996). Identification of Sonic hedgehog as a candidate gene responsible for holoprosencephaly. *Nat. Genet.* 14 353–356. 10.1038/ng1196-353 8896571

[B8] Bertrand N. Dahmane N. (2006). Sonic hedgehog signaling in forebrain development and its interactions with pathways that modify its effects. *Trends Cell Biol.* 16 597–605. 10.1016/j.tcb.2006.09.007 17030124

[B9] Borrell V. Cárdenas A. Ciceri G. Galcerán J. Flames N. Pla R. (2012). Slit/Robo signaling modulates the proliferation of central nervous system progenitors. *Neuron* 76 338–352. 10.1016/j.neuron.2012.08.003 23083737 PMC4443924

[B10] Borrell V. Reillo I. (2012). Emerging roles of neural stem cells in cerebral cortex development and evolution. *Dev. Neurobiol.* 72 955–971. 10.1002/dneu.22013 22684946

[B11] Bray S. J. (2006). Notch signalling: a simple pathway becomes complex. *Nat. Rev. Mol. Cell Biol.* 7 678–689. 10.1038/nrm2009 16921404

[B12] Briscoe S. D. Ragsdale C. W. (2018a). Homology, neocortex, and the evolution of developmental mechanisms. *Science* 362 190–193. 10.1126/science.aau3711 30309947 PMC11098553

[B13] Briscoe S. D. Ragsdale C. W. (2018b). Molecular anatomy of the alligator dorsal telencephalon. *J. Comp. Neurol.* 526 1613–1646. 10.1002/cne.24427 29520780 PMC6242290

[B14] Brose K. Bland K. S. Wang K. H. Arnott D. Henzel W. Goodman C. S. (1999). Slit proteins bind Robo receptors and have an evolutionarily conserved role in repulsive axon guidance. *Cell* 96 795–806. 10102268 10.1016/s0092-8674(00)80590-5

[B15] Bulman M. P. Kusumi K. Frayling T. M. Mckeown C. Garrett C. Lander E. S. (2000). Mutations in the human delta homologue, DLL3, cause axial skeletal defects in spondylocostal dysostosis. *Nat. Genet.* 24 438–441. 10.1038/74307 10742114

[B16] Bush G. Miyamoto A. Denault J.-B. Leduc R. Weinmaster G. (2001). Ligand-induced signaling in the absence of furin processing of notch1. *Dev. Biol.* 229 494–502. 10.1006/dbio.2000.9992 11150244

[B17] Cárdenas A. Borrell V. (2020). Molecular and cellular evolution of corticogenesis in amniotes. *Cell. Mol. Life Sci.* 77 1435–1460. 10.1007/s00018-019-03315-x 31563997 PMC11104948

[B18] Cárdenas A. Villalba A. De Juan Romero C. Picó E. Kyrousi C. Tzika A. C. (2018). Evolution of cortical neurogenesis in amniotes controlled by robo signaling levels. *Cell* 174 590.e21–606.e21. 10.1016/j.cell.2018.06.007 29961574 PMC6063992

[B19] Castro D. S. Skowronska-Krawczyk D. Armant O. Donaldson I. J. Parras C. Hunt C. (2006). Proneural bHLH and Brn proteins coregulate a neurogenic program through cooperative binding to a conserved DNA motif. *Dev. Cell* 11 831–844. 10.1016/j.devcel.2006.10.006 17141158

[B20] Chapman G. Sparrow D. B. Kremmer E. Dunwoodie S. L. (2011). Notch inhibition by the ligand Delta-Like 3 defines the mechanism of abnormal vertebral segmentation in spondylocostal dysostosis. *Hum. Mol. Genet.* 20 905–916. 10.1093/hmg/ddq529 21147753

[B21] Chen C. C. Winkler C. M. Pfenning A. R. Jarvis E. D. (2013). Molecular profiling of the developing avian telencephalon: regional timing and brain subdivision continuities. *J. Comp. Neurol.* 521 3666–3701. 10.1002/cne.23406 23818174 PMC3863995

[B22] Chen H. Thiagalingam A. Chopra H. Borges M. W. Feder J. N. Nelkin B. D. (1997). Conservation of the Drosophila lateral inhibition pathway in human lung cancer: a hairy-related protein (HES-1) directly represses achaete-scute homolog-1 expression. *Proc. Natl. Acad. Sci.* 94 5355–5360. 10.1073/pnas.94.10.5355 9144241 PMC24682

[B23] Chen N. Greenwald I. (2004). The lateral signal for LIN-12/notch in *C. elegans* vulval development comprises redundant secreted and transmembrane DSL proteins. *Dev. Cell* 6 183–192. 10.1016/s1534-5807(04)00021-8 14960273

[B24] Cinquin O. (2007). Understanding the somitogenesis clock: what’s missing? *Mech. Dev.* 124 501–517. 10.1016/j.mod.2007.06.004 17643270

[B25] Cordle J. Johnson S. Tay J. Z. Y. Roversi P. Wilkin M. B. De Madrid B. H. (2008a). A conserved face of the jagged/serrate DSL domain is involved in notch trans-activation and cis-inhibition. *Nat. Struct. Mol. Biol.* 15 849–857. 10.1038/nsmb.1457 18660822 PMC2669539

[B26] Cordle J. Redfieldz C. Stacey M. Van Der Merwe P. A. Willis A. C. Champion B. R. (2008b). Localization of the delta-like-1-binding site in human notch-1 and its modulation by calcium affinity. *J. Biol. Chem.* 283 11785–11793. 10.1074/jbc.M708424200 18296446

[B27] del Álamo D. Rouault H. Schweisguth F. (2011). Mechanism and significance of cis-inhibition in notch signalling. *Curr. Biol.* 21 R40–R47. 10.1016/j.cub.2010.10.034 21215938

[B28] Dexter J. S. (1914). The analysis of a case of continuous variation in Drosophila by a study of its linkage relations. *Am. Nat.* 48 712–758.

[B29] Dickson B. J. Gilestro G. F. (2006). Regulation of commissural axon pathfinding by slit and its Robo receptors. *Annu. Rev. Cell Dev. Biol.* 22 651–675. 10.1146/annurev.cellbio.21.090704.151234 17029581

[B30] D’souza B. Meloty-Kapella L. Weinmaster G. (2010). Canonical and non-canonical notch ligands. *Curr. Top. Dev. Biol.* 92 73–129. 10.1016/S0070-2153(10)92003-6 20816393 PMC4286395

[B31] D’souza B. Miyamoto A. Weinmaster G. (2008). The many facets of notch ligands. *Oncogene* 27 5148–5167. 10.1038/onc.2008.229 18758484 PMC2791526

[B32] Dugas-Ford J. Rowell J. J. Ragsdale C. W. (2012). Cell-type homologies and the origins of the neocortex. *Proc. Natl. Acad. Sci.* 109 16974–16979. 10.1073/pnas.1204773109 23027930 PMC3479531

[B33] Dunwoodie S. L. Clements M. Sparrow D. B. Sa X. Conlon R. A. Beddington R. S. (2002). Axial skeletal defects caused by mutation in the spondylocostal dysplasia/pudgy gene Dll3 are associated with disruption of the segmentation clock within the presomitic mesoderm. *Development* 129 1795–1806. 10.1242/dev.129.7.1795 11923214

[B34] Dunwoodie S. L. Henrique D. Harrison S. M. Beddington R. S. (1997). Mouse Dll3: a novel divergent delta gene which may complement the function of other delta homologues during early pattern formation in the mouse embryo. *Development* 124 3065–3076. 10.1242/dev.124.16.3065 9272948

[B35] Echelard Y. Epstein D. J. St-Jacques B. Shen L. Mohler J. Mcmahon J. A. (1993). Sonic hedgehog, a member of a family of putative signaling molecules, is implicated in the regulation of CNS polarity. *Cell* 75 1417–1430. 10.1016/0092-8674(93)90627-3 7916661

[B36] Eldadah Z. A. Hamosh A. Biery N. J. Montgomery R. A. Duke M. Elkins R. (2001). Familial tetralogy of fallot caused by mutation in the jagged1 gene. *Hum. Mol. Genet.* 10 163–169. 10.1093/hmg/10.2.163 11152664

[B37] Englund C. Fink A. Lau C. Pham D. Daza R. A. Bulfone A. (2005). Pax6, Tbr2, and Tbr1 are expressed sequentially by radial glia, intermediate progenitor cells, and postmitotic neurons in developing neocortex. *J. Neurosci.* 25 247–251. 10.1523/JNEUROSCI.2899-04.2005 15634788 PMC6725189

[B38] Feng L. Hatten M. E. Heintz N. (1994). Brain lipid-binding protein (BLBP): a novel signaling system in the developing mammalian CNS. *Neuron* 12 895–908. 10.1016/0896-6273(94)90341-7 8161459

[B39] Fiddes I. T. Lodewijk G. A. Mooring M. Bosworth C. M. Ewing A. D. Mantalas G. L. (2018). Human-specific NOTCH2NL genes affect notch signaling and cortical neurogenesis. *Cell* 173 1356.e–1369.e. 10.1016/j.cell.2018.03.051 29856954 PMC5986104

[B40] Fietz S. A. Kelava I. Vogt J. Wilsch-Brauninger M. Stenzel D. Fish J. L. (2010). OSVZ progenitors of human and ferret neocortex are epithelial-like and expand by integrin signaling. *Nat. Neurosci.* 13 690–699. 10.1038/nn.2553 20436478

[B41] Fischer-Zirnsak B. Segebrecht L. Schubach M. Charles P. Alderman E. Brown K. (2019). Haploinsufficiency of the notch ligand DLL1 causes variable neurodevelopmental disorders. *Am. J. Hum. Genet.* 105 631–639. 10.1016/j.ajhg.2019.07.002 31353024 PMC6731356

[B42] Fleming R. J. (1998). Structural conservation of notch receptors and ligands. *Semin. Cell Dev. Biol.* 9 599–607. 10.1006/scdb.1998.0260 9918871

[B43] Gaiano N. Nye J. S. Fishell G. (2000). Radial glial identity is promoted by notch1 signaling in the murine forebrain. *Neuron* 26 395–404. 10.1016/s0896-6273(00)81172-1 10839358

[B44] Geffers I. Serth K. Chapman G. Jaekel R. Schuster-Gossler K. Cordes R. (2007). Divergent functions and distinct localization of the notch ligands DLL1 and DLL3 in vivo. *J. Cell Biol.* 178 465–476. 10.1083/jcb.200702009 17664336 PMC2064846

[B45] Gertz C. C. Kriegstein A. R. (2015). Neuronal migration dynamics in the developing ferret cortex. *J. Neurosci.* 35 14307–14315. 10.1523/JNEUROSCI.2198-15.2015 26490868 PMC4683689

[B46] Geschwind D. H. Rakic P. (2013). Cortical evolution: judge the brain by its cover. *Neuron* 80 633–647. 10.1016/j.neuron.2013.10.045 24183016 PMC3922239

[B47] Go M. J. Eastman D. S. Artavanis-Tsakonas S. (1998). Cell proliferation control by notch signaling in Drosophila development. *Development* 125 2031–2040. 10.1242/dev.125.11.2031 9570768

[B48] Goffinet A. M. Daumerie C. Langerwerf B. Pieau C. (1986). Neurogenesis in reptilian cortical structures: 3H-thymidine autoradiographic analysis. *J. Comp. Neurol.* 243 106–116. 10.1002/cne.902430109 3950076

[B49] Gordon W. R. Vardar-Ulu D. Histen G. Sanchez-Irizarry C. Aster J. C. Blacklow S. C. (2007). Structural basis for autoinhibition of notch. *Nat. Struct. Mol. Biol.* 14 295–300. 10.1038/nsmb1227 17401372

[B50] Govindan S. Jabaudon D. (2017). Coupling progenitor and neuronal diversity in the developing neocortex. *FEBS Lett.* 591 3960–3977. 10.1002/1873-3468.12846 28895133

[B51] Guillemot F. (2005). Cellular and molecular control of neurogenesis in the mammalian telencephalon. *Curr. Opin. Cell Biol.* 17 639–647. 10.1016/j.ceb.2005.09.006 16226447

[B52] Hansen D. V. Lui J. H. Parker P. R. Kriegstein A. R. (2010). Neurogenic radial glia in the outer subventricular zone of human neocortex. *Nature* 464 554–561. 10.1038/nature08845 20154730

[B53] Hansson E. M. Lanner F. Das D. Mutvei A. Marklund U. Ericson J. (2010). Control of notch-ligand endocytosis by ligand-receptor interaction. *J. Cell Sci.* 123 2931–2942. 10.1242/jcs.073239 20720151

[B54] Hatakeyama J. Bessho Y. Katoh K. Ookawara S. Fujioka M. Guillemot F. (2004). Hes genes regulate size, shape and histogenesis of the nervous system by control of the timing of neural stem cell differentiation. *Development* 131 5539–5550. 10.1242/dev.01436 15496443

[B55] Henderson S. T. Gao D. Christensen S. Kimble J. (1997). Functional domains of LAG-2, a putative signaling ligand for LIN-12 and GLP-1 receptors in *Caenorhabditis elegans*. *Mol. Biol. Cell* 8 1751–1762. 10.1091/mbc.8.9.1751 9307971 PMC305734

[B56] Henderson S. T. Gao D. Lambie E. J. Kimble J. (1994). lag-2 may encode a signaling ligand for the GLP-1 and LIN-12 receptors of C. elegans. *Development* 120 2913–2924. 10.1242/dev.120.10.2913 7607081

[B57] Herman A. Rhyner A. Devine P. Marrelli S. Bruneau B. Wythe J. (2018). A novel reporter allele for monitoring Dll4 expression within the embryonic and adult mouse. *Biol. Open* 7:bio026799. 10.1242/bio.026799 29437553 PMC5898260

[B58] His W. (1889). *Die Neuroblasten Und Deren Entstehung im Embryonalen Mark.* Leipzig: S. Hirzel.

[B59] Holley S. A. Geisler R. Nüsslein-Volhard C. (2000). Control of her1 expression during zebrafish somitogenesis by a delta-dependent oscillator and an independent wave-front activity. *Genes Dev.* 14 1678–1690. 10887161 PMC316735

[B60] Hubaud A. Pourquié O. (2014). Signalling dynamics in vertebrate segmentation. *Nat. Rev. Mol. Cell Biol.* 15 709–721. 10.1038/nrm3891 25335437

[B61] Huesa G. Anadón R. Folgueira M. Yáñez J. (2009). “Evolution of the Pallium in Fishes,” in *Encyclopedia of Neuroscience*, eds Binder M. D. Hirokawa N. Windhorst U. (Berlin: Springer), 1400–1404. 10.1007/978-3-540-29678-2_3166

[B62] Imayoshi I. Isomura A. Harima Y. Kawaguchi K. Kori H. Miyachi H. (2013). Oscillatory control of factors determining multipotency and fate in mouse neural progenitors. *Science* 342 1203–1208. 10.1126/science.1242366 24179156

[B63] Jarriault S. Bail O. L. Hirsinger E. Pourquié O. Logeat F. Strong C. F. (1998). Delta-1 activation of notch-1 signaling results in HES-1 transactivation. *Mol. Cell. Biol.* 18 7423–7431. 10.1128/MCB.18.12.7423 9819428 PMC109323

[B64] Jarvis E. D. (2009). “Evolution of the Pallium in Birds and Reptiles,” in *Encyclopedia of Neuroscience*, eds Binder M. D. Hirokawa N. Windhorst U. (Berlin: Springer), 1390–1400. 10.1007/978-3-540-29678-2_3165

[B65] Kageyama R. Niwa Y. Isomura A. González A. Harima Y. (2012). Oscillatory gene expression and somitogenesis. *Wiley Interdiscip. Rev. Dev. Biol.* 1 629–641. 10.1002/wdev.46 23799565

[B66] Karten H. J. (1991). Homology and evolutionary origins of the ‘neocortex’. *Brain Behav. Evol.* 38 264–272. 10.1159/000114393 1777808

[B67] Karten H. J. Shimizu T. (1989). The origins of neocortex: connections and lamination as distinct events in evolution. *J. Cogn. Neurosci.* 1 291–301. 10.1162/jocn.1989.1.4.291 23971981

[B68] Kawaguchi D. Yoshimatsu T. Hozumi K. Gotoh Y. (2008). Selection of differentiating cells by different levels of delta-like 1 among neural precursor cells in the developing mouse telencephalon. *Development* 135 3849–3858. 10.1242/dev.024570 18997111

[B69] Kobayashi T. Kageyama R. (2011). Hes1 oscillations contribute to heterogeneous differentiation responses in embryonic stem cells. *Genes* 2 219–228. 10.3390/genes2010219 24710146 PMC3924840

[B70] Kobayashi T. Mizuno H. Imayoshi I. Furusawa C. Shirahige K. Kageyama R. (2009). The cyclic gene Hes1 contributes to diverse differentiation responses of embryonic stem cells. *Genes Dev.* 23 1870–1875. 10.1101/gad.1823109 19684110 PMC2725939

[B71] Komada M. (2012). Sonic hedgehog signaling coordinates the proliferation and differentiation of neural stem/progenitor cells by regulating cell cycle kinetics during development of the neocortex. *Congenit. Anom.* 52 72–77. 10.1111/j.1741-4520.2012.00368.x 22639991

[B72] Komada M. Saitsu H. Kinboshi M. Miura T. Shiota K. Ishibashi M. (2008). Hedgehog signaling is involved in development of the neocortex. *Development* 135 2717–2727. 10.1242/dev.015891 18614579

[B73] Komatsu H. Chao M. Y. Larkins-Ford J. Corkins M. E. Somers G. A. Tucey T. (2008). OSM-11 facilitates LIN-12 notch signaling during *Caenorhabditis elegans* vulval development. *PLoS Biol.* 6:e196. 10.1371/journal.pbio.0060196 18700817 PMC2504490

[B74] Koo B.-K. Lim H.-S. Song R. Yoon M.-J. Yoon K.-J. Moon J.-S. (2005). Mind bomb 1 is essential for generating functional notch ligands to activate notch. *Development* 132 3459–3470. 10.1242/dev.01922 16000382

[B75] Kopan R. Ilagan M. X. G. (2009). The canonical notch signaling pathway: unfolding the activation mechanism. *Cell* 137 216–233. 10.1016/j.cell.2009.03.045 19379690 PMC2827930

[B76] Kusumi K. Sun E. S. Kerrebrock A. W. Bronson R. T. Chi D. C. Bulotsky M. S. (1998). The mouse pudgy mutation disrupts Delta homologue Dll3 and initiation of early somite boundaries. *Nat. Genet.* 19 274–278. 10.1038/961 9662403

[B77] Ladi E. Nichols J. T. Ge W. Miyamoto A. Yao C. Yang L. T. (2005). The divergent DSL ligand Dll3 does not activate Notch signaling but cell autonomously attenuates signaling induced by other DSL ligands. *J. Cell Biol.* 170 983–992. 10.1083/jcb.200503113 16144902 PMC2171428

[B78] Le Borgne R. Bardin A. Schweisguth F. (2005). The roles of receptor and ligand endocytosis in regulating notch signaling. *Development* 132 1751–1762. 10.1242/dev.01789 15790962

[B79] LeBon L. Lee T. V. Sprinzak D. Jafar-Nejad H. Elowitz M. B. (2014). Fringe proteins modulate notch-ligand cis and trans interactions to specify signaling states. *eLife* 3:e02950. 10.7554/eLife.02950 25255098 PMC4174579

[B80] Levitt P. Rakic P. (1980). Immunoperoxidase localization of glial fibrillary acidic protein in radial glial cells and astrocytes of the developing rhesus monkey brain. *J. Comp. Neurol.* 193 815–840. 10.1002/cne.901930316 7002963

[B81] Liao B.-K. Jörg D. J. Oates A. C. (2016). Faster embryonic segmentation through elevated delta-notch signalling. *Nat. Commun.* 7 1–12. 10.1038/ncomms11861 27302627 PMC4912627

[B82] Llinares-Benadero C. Borrell V. (2019). Deconstructing cortical folding: genetic, cellular and mechanical determinants. *Nat. Rev. Neurosci.* 20 161–176. 10.1038/s41583-018-0112-2 30610227

[B83] Lubman O. Y. Ilagan M. X. G. Kopan R. Barrick D. (2007). Quantitative dissection of the notch: CSL interaction: insights into the notch-mediated transcriptional switch. *J. Mol. Biol.* 365 577–589. 10.1016/j.jmb.2006.09.071 17070841 PMC1851696

[B84] Lui J. H. Hansen D. V. Kriegstein A. R. (2011). Development and evolution of the human neocortex. *Cell* 146 18–36. 21729779 10.1016/j.cell.2011.06.030 PMC3610574

[B85] Lukaszewicz A. Savatier P. Cortay V. Giroud P. Huissoud C. Berland M. (2005). G1 phase regulation, area-specific cell cycle control, and cytoarchitectonics in the primate cortex. *Neuron* 47 353–364. 10.1016/j.neuron.2005.06.032 16055060 PMC1890568

[B86] Luzzati F. (2015). A hypothesis for the evolution of the upper layers of the neocortex through co-option of the olfactory cortex developmental program. *Front. Neurosci.* 9:162. 10.3389/fnins.2015.00162 26029038 PMC4429232

[B87] Malatesta P. Hartfuss E. Gotz M. (2000). Isolation of radial glial cells by fluorescent-activated cell sorting reveals a neuronal lineage. *Development* 127 5253–5263. 10.1242/dev.127.24.5253 11076748

[B88] Mara A. Schroeder J. Chalouni C. Holley S. A. (2007). Priming, initiation and synchronization of the segmentation clock by deltaD and deltaC. *Nat. Cell Biol.* 9 523–530. 10.1038/ncb1578 17417625

[B89] Mase S. Shitamukai A. Wu Q. Morimoto M. Gridley T. Matsuzaki F. (2021). notch1 and notch2 collaboratively maintain radial glial cells in mouse neurogenesis. *Neurosci. Res.* 170 122–132. 10.1016/j.neures.2020.11.007 33309869

[B90] Matsuda M. Yamanaka Y. Uemura M. Osawa M. Saito M. K. Nagahashi A. (2020). Recapitulating the human segmentation clock with pluripotent stem cells. *Nature* 580 124–129. 10.1038/s41586-020-2144-9 32238941

[B91] Mehta B. Bhat K. M. (2001). Slit signaling promotes the terminal asymmetric division of neural precursor cells in the Drosophila CNS. *Development* 128 3161–3168. 10.1242/dev.128.16.3161 11688564

[B92] Miyata T. Kawaguchi A. Saito K. Kawano M. Muto T. Ogawa M. (2004). Asymmetric production of surface-dividing and non-surface-dividing cortical progenitor cells. *Development* 131 3133–3145. 10.1242/dev.01173 15175243

[B93] Miyoshi G. Fishell G. (2012). Dynamic FoxG1 expression coordinates the integration of multipolar pyramidal neuron precursors into the cortical plate. *Neuron* 74 1045–1058. 10.1016/j.neuron.2012.04.025 22726835 PMC3653132

[B94] Mizutani K.-I. Yoon K. Dang L. Tokunaga A. Gaiano N. (2007). Differential notch signalling distinguishes neural stem cells from intermediate progenitors. *Nature* 449 351–355. 10.1038/nature06090 17721509

[B95] Mohler J. (1988). Requirements for hedgehog, a segmental polarity gene, in patterning larval and adult cuticle of Drosophila. *Genetics* 120 1061–1072. 10.1093/genetics/120.4.1061 3147217 PMC1203569

[B96] Morgan T. H. (1911). The origin of nine wing mutations in drosophila. *Science* 33 496–499. 10.1126/science.33.848.496 17774436

[B97] Morgan T. H. (1917). The theory of the gene. *Am. Nat.* 51 513–544.

[B98] Mueller T. Wullimann M. F. (2003). Anatomy of neurogenesis in the early zebrafish brain. *Dev. Brain Res.* 140 137–155. 10.1016/s0165-3806(02)00583-7 12524185

[B99] Murone M. Rosenthal A. De Sauvage F. J. (1999). Sonic hedgehog signaling by the patched–smoothened receptor complex. *Curr. Biol.* 9 76–84. 10.1016/s0960-9822(99)80018-9 10021362

[B100] Nandagopal N. Santat L. A. Elowitz M. B. (2019). Cis-activation in the notch signaling pathway. *eLife* 8:e37880. 10.7554/eLife.37880 30628888 PMC6345567

[B101] Nelson B. R. Hodge R. D. Bedogni F. Hevner R. F. (2013). Dynamic interactions between intermediate neurogenic progenitors and radial glia in embryonic mouse neocortex: potential role in Dll1-Notch signaling. *J. Neurosci.* 33 9122–9139. 10.1523/JNEUROSCI.0791-13.2013 23699523 PMC3716275

[B102] Noctor S. C. Flint A. C. Weissman T. A. Dammerman R. S. Kriegstein A. R. (2001). Neurons derived from radial glial cells establish radial units in neocortex. *Nature* 409 714–720. 10.1038/35055553 11217860

[B103] Noctor S. C. Flint A. C. Weissman T. A. Wong W. S. Clinton B. K. Kriegstein A. R. (2002). Dividing precursor cells of the embryonic cortical ventricular zone have morphological and molecular characteristics of radial glia. *J. Neurosci.* 22 3161–3173. 10.1523/JNEUROSCI.22-08-03161.2002 11943818 PMC6757532

[B104] Noctor S. C. Martínez-Cerdeño V. Ivic L. Kriegstein A. R. (2004). Cortical neurons arise in symmetric and asymmetric division zones and migrate through specific phases. *Nat. Neurosci.* 7 136–144. 10.1038/nn1172 14703572

[B105] Nofziger D. Miyamoto A. Lyons K. M. Weinmaster G. (1999). Notch signaling imposes two distinct blocks in the differentiation of C2C12 myoblasts. *Development* 126 1689–1702. 10.1242/dev.126.8.1689 10079231

[B106] Nomura T. Gotoh H. Ono K. (2013). Changes in the regulation of cortical neurogenesis contribute to encephalization during amniote brain evolution. *Nat. Commun.* 4:2206. 10.1038/ncomms3206 23884180

[B107] Nomura T. Ohtaka-Maruyama C. Kiyonari H. Gotoh H. Ono K. (2020). Changes in wnt-dependent neuronal morphology underlie the anatomical diversification of neocortical homologs in amniotes. *Cell Rep.* 31:107592. 10.1016/j.celrep.2020.107592 32375034

[B108] Nüsslein-Volhard C. Wieschaus E. (1980). Mutations affecting segment number and polarity in Drosophila. *Nature* 287 795–801. 10.1038/287795a0 6776413

[B109] Ochi S. Imaizumi Y. Shimojo H. Miyachi H. Kageyama R. (2020). Oscillatory expression of Hes1 regulates cell proliferation and neuronal differentiation in the embryonic brain. *Development* 147:dev182204. 10.1242/dev.182204 32094111

[B110] Ohtsuka T. Kageyama R. (2021a). Dual activation of Shh and Notch signaling induces dramatic enlargement of neocortical surface area. *Neurosci. Res.* Online ahead of print., 10.1016/j.neures.2021.09.006, 34600946

[B111] Ohtsuka T. Kageyama R. (2021b). Hes1 overexpression leads to expansion of embryonic neural stem cell pool and stem cell reservoir in the postnatal brain. *Development* 148:dev189191. 10.1242/dev.189191 33531431

[B112] Palmeirim I. Henrique D. Ish-Horowicz D. Pourquié O. (1997). Avian hairy gene expression identifies a molecular clock linked to vertebrate segmentation and somitogenesis. *Cell* 91 639–648. 10.1016/s0092-8674(00)80451-1 9393857

[B113] Purow B. W. Haque R. M. Noel M. W. Su Q. Burdick M. J. Lee J. (2005). Expression of notch-1 and its ligands, delta-like-1 and jagged-1, is critical for glioma cell survival and proliferation. *Cancer Res.* 65 2353–2363. 10.1158/0008-5472.CAN-04-1890 15781650

[B114] Rakic P. (1972). Mode of cell migration to the superficial layers of fetal monkey neocortex. *J. Comp. Neurol.* 145 61–83. 10.1002/cne.901450105 4624784

[B115] Rakic P. (1974). Neurons in rhesus monkey visual cortex: systematic relation between time of origin and eventual disposition. *Science* 183 425–427. 10.1126/science.183.4123.425 4203022

[B116] Ramon y Cajal S Azoulay L. (1955). *Histologie du Systeme Nerveux de L’homme et Des Vertebres.* Paris: Maloine.

[B117] Rao Z. Handford P. Mayhew M. Knott V. Brownlee G. G. Stuartz D. (1995). The structure of a Ca2+-binding epidermal growth factor-like domain: its role in protein-protein interactions. *Cell* 82 131–141. 10.1016/0092-8674(95)90059-4 7606779

[B118] Raya Á Kawakami Y. Rodríguez-Esteban C. Ibañes M. Rasskin-Gutman D. Rodríguez-León J. (2004). Notch activity acts as a sensor for extracellular calcium during vertebrate left–right determination. *Nature* 427 121–128. 10.1038/nature02190 14712268

[B119] Reillo I. De Juan Romero C. García-Cabezas M. Borrell V. (2011). A role for intermediate radial glia in the tangential expansion of the mammalian cerebral cortex. *Cereb. Cortex* 21 1674–1694. 10.1093/cercor/bhq238 21127018

[B120] Roelink H. Porter J. Chiang C. Tanabe Y. Chang D. Beachy P. (1995). Floor plate and motor neuron induction by different concentrations of the amino-terminal cleavage product of sonic hedgehog autoproteolysis. *Cell* 81 445–455. 10.1016/0092-8674(95)90397-6 7736596

[B121] Roessler E. Belloni E. Gaudenz K. Jay P. Berta P. Scherer S. W. (1996). Mutations in the human Sonic Hedgehog gene cause holoprosencephaly. *Nat. Genet.* 14 357–360. 10.1038/ng1196-357 8896572

[B122] Ronchini C. Capobianco A. J. (2001). Induction of cyclin D1 transcription and CDK2 activity by Notchic: implication for cell cycle disruption in transformation by Notchic. *Mol. Cell. Biol.* 21 5925–5934. 10.1128/MCB.21.17.5925-5934.2001 11486031 PMC87311

[B123] Saade M. Gutiérrez-Vallejo I. Le Dréau G. Rabadán M. A. Miguez D. G. Buceta J. (2013). Sonic hedgehog signaling switches the mode of division in the developing nervous system. *Cell Rep.* 4 492–503. 10.1016/j.celrep.2013.06.038 23891002

[B124] Sang L. Coller H. A. Roberts J. M. (2008). Control of the reversibility of cellular quiescence by the transcriptional repressor HES1. *Science* 321 1095–1100. 10.1126/science.1155998 18719287 PMC2721335

[B125] Shawber C. Nofziger D. Hsieh J. Lindsell C. Bogler O. Hayward D. (1996). Notch signaling inhibits muscle cell differentiation through a CBF1-independent pathway. *Development* 122 3765–3773. 10.1242/dev.122.12.3765 9012498

[B126] Sherwood D. R. McClay D. R. (1997). Identification and localization of a sea urchin Notch homologue: insights into vegetal plate regionalization and Notch receptor regulation. *Development* 124 3363–3374. 10.1242/dev.124.17.3363 9310331

[B127] Shibata T. Yamada K. Watanabe M. Ikenaka K. Wada K. Tanaka K. (1997). Glutamate transporter GLAST is expressed in the radial glia-astrocyte lineage of developing mouse spinal cord. *J. Neurosci.* 17 9212–9219. 10.1523/JNEUROSCI.17-23-09212.1997 9364068 PMC6573593

[B128] Shikata Y. Okada T. Hashimoto M. Ellis T. Matsumaru D. Shiroishi T. (2011). Ptch1-mediated dosage-dependent action of Shh signaling regulates neural progenitor development at late gestational stages. *Dev. Biol.* 349 147–159. 10.1016/j.ydbio.2010.10.014 20969845

[B129] Shimizu K. Chiba S. Kumano K. Hosoya N. Takahashi T. Kanda Y. (1999). Mouse jagged1 physically interacts with notch2 and other notch receptors: assessment by quantitative methods. *J. Biol. Chem.* 274 32961–32969. 10.1074/jbc.274.46.32961 10551863

[B130] Shimojo H. Isomura A. Ohtsuka T. Kori H. Miyachi H. Kageyama R. (2016). Oscillatory control of Delta-like1 in cell interactions regulates dynamic gene expression and tissue morphogenesis. *Genes Dev.* 30 102–116. 10.1101/gad.270785.115 26728556 PMC4701973

[B131] Shimojo H. Ohtsuka T. Kageyama R. (2008). Oscillations in notch signaling regulate maintenance of neural progenitors. *Neuron* 58 52–64. 10.1016/j.neuron.2008.02.014 18400163

[B132] Smart I. H. Dehay C. Giroud P. Berland M. Kennedy H. (2002). Unique morphological features of the proliferative zones and postmitotic compartments of the neural epithelium giving rise to striate and extrastriate cortex in the monkey. *Cereb. Cortex* 12 37–53. 10.1093/cercor/12.1.37 11734531 PMC1931430

[B133] Smithers L. Haddon C. Jiang Y.-J. Lewis J. (2000). Sequence and embryonic expression of deltaC in the zebrafish. *Mech. Dev.* 90 119–123. 10.1016/s0925-4773(99)00231-2 10585570

[B134] Sprinzak D. Blacklow S. C. (2021). Biophysics of notch signaling. *Annu. Rev. Biophys.* 50 157–189. 10.1146/annurev-biophys-101920-082204 33534608 PMC8105286

[B135] Sprinzak D. Lakhanpal A. Lebon L. Santat L. A. Fontes M. E. Anderson G. A. (2010). Cis-interactions between notch and delta generate mutually exclusive signalling states. *Nature* 465 86–90. 10.1038/nature08959 20418862 PMC2886601

[B136] Sueda R. Imayoshi I. Harima Y. Kageyama R. (2019). High Hes1 expression and resultant Ascl1 suppression regulate quiescent vs. active neural stem cells in the adult mouse brain. *Genes Dev.* 33 511–523. 10.1101/gad.323196.118 30862661 PMC6499325

[B137] Suzuki I. Hirata T. (2014). A common developmental plan for neocortical gene-expressing neurons in the pallium of the domestic chicken Gallus gallus domesticus and the Chinese softshell turtle *Pelodiscus sinensis*. *Front. Neuroanat.* 8:20. 10.3389/fnana.2014.00020 24778607 PMC3985024

[B138] Suzuki I. K. Gacquer D. Van Heurck R. Kumar D. Wojno M. Bilheu A. (2018). Human-specific NOTCH2NL genes expand cortical neurogenesis through delta/notch regulation. *Cell* 173 1370.e16–1384.e16. 10.1016/j.cell.2018.03.067 29856955 PMC6092419

[B139] Suzuki I. K. Kawasaki T. Gojobori T. Hirata T. (2012). The temporal sequence of the mammalian neocortical neurogenetic program drives mediolateral pattern in the chick pallium. *Dev. Cell* 22 863–870. 10.1016/j.devcel.2012.01.004 22424929

[B140] Takano A. Zochi R. Hibi M. Terashima T. Katsuyama Y. (2011). Function of strawberry notch family genes in the zebrafish brain development. *Kobe J. Med. Sci.* 56 E220–E230. 21937870

[B141] Tax F. E. Yeargers J. J. Thomas J. H. (1994). Sequence of *C. elegans* lag-2 reveals a cell-signalling domain shared with delta and serrate of Drosophila. *Nature* 368 150–154. 10.1038/368150a0 8139658

[B142] Tekendo-Ngongang C. Muenke M. Kruszka P. (1993). “Holoprosencephaly overview,” in *GeneReviews(§)*, eds Adam M. P. Ardinger H. H. Pagon R. A. Wallace S. E. Bean L. J. H. Mirzaa G. (Seattle, WA: University of Washington, Seattle).

[B143] Tomita K. Hattori M. Nakamura E. Nakanishi S. Minato N. Kageyama R. (1999). The bHLH gene Hes1 is essential for expansion of early T cell precursors. *Genes Dev.* 13 1203–1210. 10.1101/gad.13.9.1203 10323870 PMC316958

[B144] Tosches M. A. Yamawaki T. M. Naumann R. K. Jacobi A. A. Tushev G. Laurent G. (2018). Evolution of pallium, hippocampus, and cortical cell types revealed by single-cell transcriptomics in reptiles. *Science* 360 881–888. 10.1126/science.aar4237 29724907

[B145] Tozer S. Baek C. Fischer E. Goiame R. Morin X. (2017). Differential routing of mindbomb1 via centriolar satellites regulates asymmetric divisions of neural progenitors. *Neuron* 93, 542–551.e4. 10.1016/j.neuron.2016.12.042 28132826

[B146] Tsai H. Hardisty R. E. Rhodes C. Kiernan A. E. Roby P. Tymowska-Lalanne Z. (2001). The mouse slalom mutant demonstrates a role for Jagged1 in neuroepithelial patterning in the organ of Corti. *Hum. Mol. Genet.* 10 507–512. 10.1093/hmg/10.5.507 11181574

[B147] Turnpenny P. D. Alman B. Cornier A. S. Giampietro P. F. Offiah A. Tassy O. (2007). Abnormal vertebral segmentation and the notch signaling pathway in man. *Dev. Dyn.* 236 1456–1474. 10.1002/dvdy.21182 17497699

[B148] Warthen D. Moore E. Kamath B. Morrissette J. Sanchez P. Piccoli D. (2006). Jagged1 (JAG1) mutations in Alagille syndrome: increasing the mutation detection rate. *Hum. Mutat.* 27 436–443. 10.1002/humu.20310 16575836

[B149] Weng A. P. Millholland J. M. Yashiro-Ohtani Y. Arcangeli M. L. Lau A. Wai C. (2006). c-Myc is an important direct target of notch1 in T-cell acute lymphoblastic leukemia/lymphoma. *Genes Dev.* 20 2096–2109. 10.1101/gad.1450406 16847353 PMC1536060

[B150] Wickström M. Dyberg C. Shimokawa T. Milosevic J. Baryawno N. Fuskevåg O. M. (2013). Targeting the hedgehog signal transduction pathway at the level of GLI inhibits neuroblastoma cell growth in vitro and in vivo. *Int. J. Cancer* 132 1516–1524. 10.1002/ijc.27820 22949014

[B151] Wright G. J. Giudicelli F. Soza-Ried C. Hanisch A. Ariza-Mcnaughton L. Lewis J. (2011). DeltaC and DeltaD interact as notch ligands in the zebrafish segmentation clock. *Development* 138 2947–2956. 10.1242/dev.066654 21653612

[B152] Wullimann M. F. (2009). Secondary neurogenesis and telencephalic organization in zebrafish and mice: a brief review. *Integr. Zool.* 4 123–133. 10.1111/j.1749-4877.2008.00140.x 21392282

[B153] Wullimann M. F. Mueller T. (2004). Teleostean and mammalian forebrains contrasted: evidence from genes to behavior. *J. Comp. Neurol.* 475 143–162. 10.1002/cne.20183 15211457

[B154] Yamamoto K. Bloch S. Vernier P. (2017). New perspective on the regionalization of the anterior forebrain in Osteichthyes. *Dev. Growth Differ.* 59 175–187. 10.1111/dgd.12348 28470718 PMC11520958

[B155] Yoon K.-J. Koo B.-K. Im S.-K. Jeong H.-W. Ghim J. Kwon M.-C. (2008). Mind bomb 1-expressing intermediate progenitors generate notch signaling to maintain radial glial cells. *Neuron* 58 519–531. 10.1016/j.neuron.2008.03.018 18498734

[B156] Zeier H. Karten H. J. (1971). The archistriatum of the pigeon: organization of afferent and efferent connections. *Brain Res.* 31 313–326. 10.1016/0006-8993(71)90185-5 5569153

[B157] Zolkiewska A. (2008). ADAM proteases: ligand processing and modulation of the notch pathway. *Cell. Mol. Life Sci.* 65 2056–2068. 10.1007/s00018-008-7586-4 18344021 PMC2674646

